# Chemoprotective effects of inositol hexaphosphate against cyclophosphamide-induced testicular damage in rats

**DOI:** 10.1038/s41598-020-68608-9

**Published:** 2020-07-28

**Authors:** Maha I. Alkhalaf, Wafa S. Alansari, Fawzia A. Alshubaily, Afnan M. Alnajeebi, Areej A. Eskandrani, Manal A. Tashkandi, Nouf A. Babteen

**Affiliations:** 1 Biochemistry Department, Faculty of Science, University of Jeddah, Jeddah, Saudi Arabia; 2 Biochemistry Department, Faculty of Science, King Abdulaziz University, Jeddah, Saudi Arabia; 3 Chemistry Department, Faculty of Science, Taibah University, Medina, Saudi Arabia

**Keywords:** Biochemistry, Cancer

## Abstract

Cyclophosphamide (CP) is commonly used as an anticancer agent but has been associated with high toxicity in several animal organs, including the testes. Inositol hexaphosphate (IP6) is a polyphosphorylated carbohydrate that is present in foods with high fibre contents and has a wide range of essential physiological and pathological activities. Thus, we estimated the defensive effects of IP6 against CP-related testicular toxicity in rats. Sperm counts, motilities, viabilities and abnormalities and levels of testosterone, luteinising hormone and follicle-stimulating hormone were evaluated. Testicle specimens were also processed for histological and biochemical analyses, including determinations of malondialdehyde, nitric oxide, total antioxidant capacity, alkaline phosphatase, acid phosphatase, gamma glutamyl transferase, ß-glucuronidase, c-reactive protein, monocyte chemoattractant protein and leukotriene-4 and in comet assays. CP treatments were associated with deleterious histopathological, biochemical and genetic changes in rat testicles, and these were ameliorated by IP6 supplements in drinking water.

## Introduction

Gonadal toxicity of chemotherapy drugs, particularly alkylating agents, is correlated with types of anti-inflammatory agents, types of chemotherapy agents, total doses, durations of treatment and individual sensitivities. Cyclophosphamide (CP), or (RS)-N, N-bis (2-chloroethyl)-1,3,2-oxazaphosphinan-2-amine 2-oxide, is a cytostatic alkylating agent that was derived from bis-b-chloroethylamine^1^. CP has since become one of the most commonly used human and veterinary anti-tumour and immunosuppressive medicines^2^. Although its anti-tumour and immunosuppressive properties have led to extensive use in the management of tumours, such as breast and prostate carcinomas, CP has a variety of reproductive side effects^3^. CP also causes gastrointestinal side effects that present as anorexia, vomiting, nausea, haemorrhagic colitis, diarrhoea, stomatitis, acute pancreatitis, jaundice-related hepatic damage and elevated liver transaminase in humans^4^. The ensuing ultrastructural changes are correlated with clinical symptoms and irregular laboratory data^5^. Moreover, following hepatic metabolic activation, CP is converted to 4-hydroxy Cyclophosphamide, which transforms into the cytotoxic metabolites acrolein and phosphoramide mustard. These cytotoxic metabolites form covalent bonds with DNA and proteins and thereby contribute to cell death following enzyme activation^6^. The anti-cancer effects of CP are correlated with phosphoramide mustard production, although acrolein is correlated with toxic side effects. These negative biochemical responses lead to oxidative stress and reduced fertility in patients. Moreover, irregular cellular processes, necrosis and cell death follow interactions between acrolein and DNA^7^.

Infertility is a major challenge among younger male CP-treated patients, and in most studies of cell proliferation, CP is cytotoxic in rapidly dividing cells, reflecting the harmful effects of this drug^8^. CP treatments contribute to male fertility by increasing frequencies of oligospermia and azoospermia^9^. Hence, interactions of CP with rapidly proliferating tissues are core to its therapeutic properties and toxic effects. Because of the effects of CP on rapidly dividing cells, testicle tissues are highly sensitive to this agent^10^, reflecting accumulation of reactive oxygen species (ROS) and increased absorption of polyunsaturated fatty acids, which play major roles in protection against oxidative damage^11^. Effects of CP on the pituitary–gonadal axis have also been suggested to reduce rates of spermatogenesis and oogenesis^12^.

In addition to cancer patients, CP risks are relevant to pharmacists and healthcare workers, who can be exposed to the drug during manufacture and delivery. Thus, new therapeutic approaches are still required to manage the reproductive toxicity of CP. Several medical plants reportedly mitigate the harmful effects of CP on certain reproductive parameters in rats^13^.

Inositol hexaphosphate (IP6) comprises a simple carbohydrate ring with phosphate groups on all six carbon atoms. This compound is the main form of inositol found in foods, comprising 1%–5% of the dry weights of most cereals, nuts, oleaginous seeds, legumes and grains. Several health benefits of IP6 from rice bran have been reported to possess various medicinal properties^14^. Hence, IP6 supplements may provide significant health benefits due to inhibition of renal calculus, cardiovascular damage, certain cancrs and osteoporosis. Because of the dephosphorylation of IP6 by phosphatases and de novo synthesis in cells, the inositol phosphate chemistry is complicated by multiple isomers in vivo^15^. Dietary IP6 is primarily digested by bacterial phytases and phosphatases in the intestine, and these microbiota release myo-ins and other phosphates of inositol. Although a controversial suggestion, variable fractions of dietary IP6 are immediately absorbed and recovered in plasma and urine^16^. Herein, we investigated defensive roles of IP6 against the testicular toxicity of CP Male albino rats.

## Results

### Effects of CP and IP6 treatments on sperm characteristics in rats

**Figure 1:**
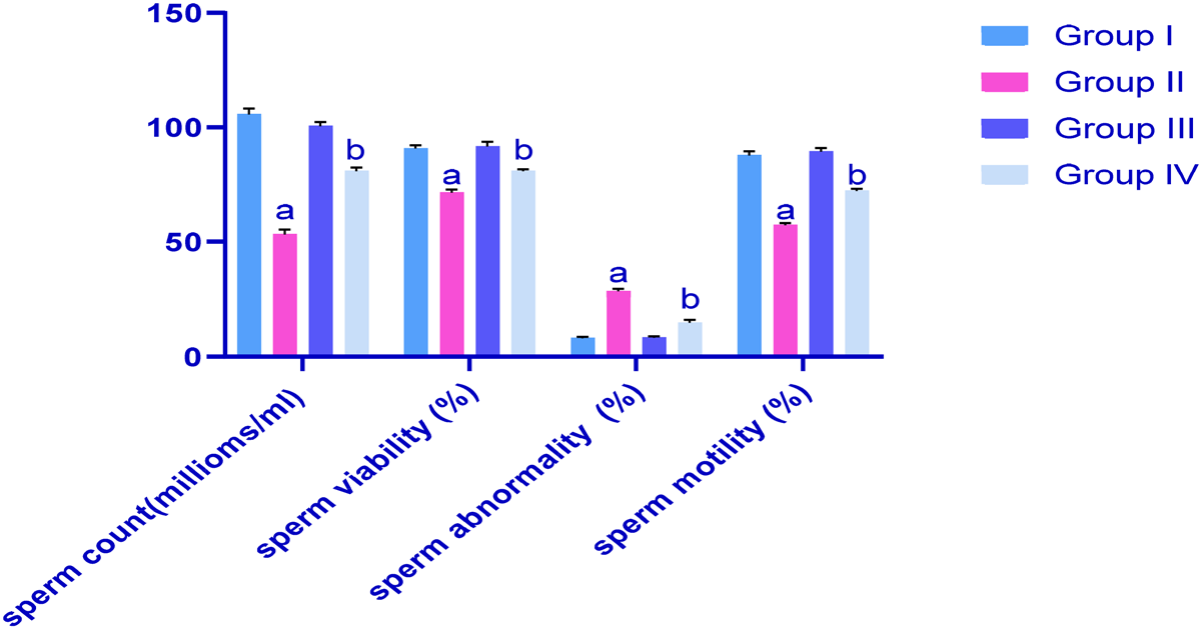
Effects of cyclophosphamide (CP) and/or inositol 6 phosphate (IP6) treatments on sperm characteristics in rats; G1, control; G2, CP-treated group; G3, IP6-treated group; G4: CP + IP6-treated group; ^a^significant differences between G1 and G3 (*p* < 0.05); ^s^ignificant differences with G2 (*p* < 0.05); data are presented as means ± standard errors of the mean (SEM; N = 8), and differences were considered significant when *p* < 0.05.

In Fig. 1, we demonstrate that CP-induced testicular injury is indicated by significant reductions (*p* < 0.05) in sperm counts, sperm viabilities and sperm motilities and by significant increases (*p* < 0.05) in sperm abnormalities, as compared with the healthy control group. Concomitant treatments with IP6 led to improvements in all these parameters, as compared with those in G2.

### Determination of testosterone, luteinising hormone (LH) and follicle-stimulating hormone (FSH) levels in rats

**Figure 2:**
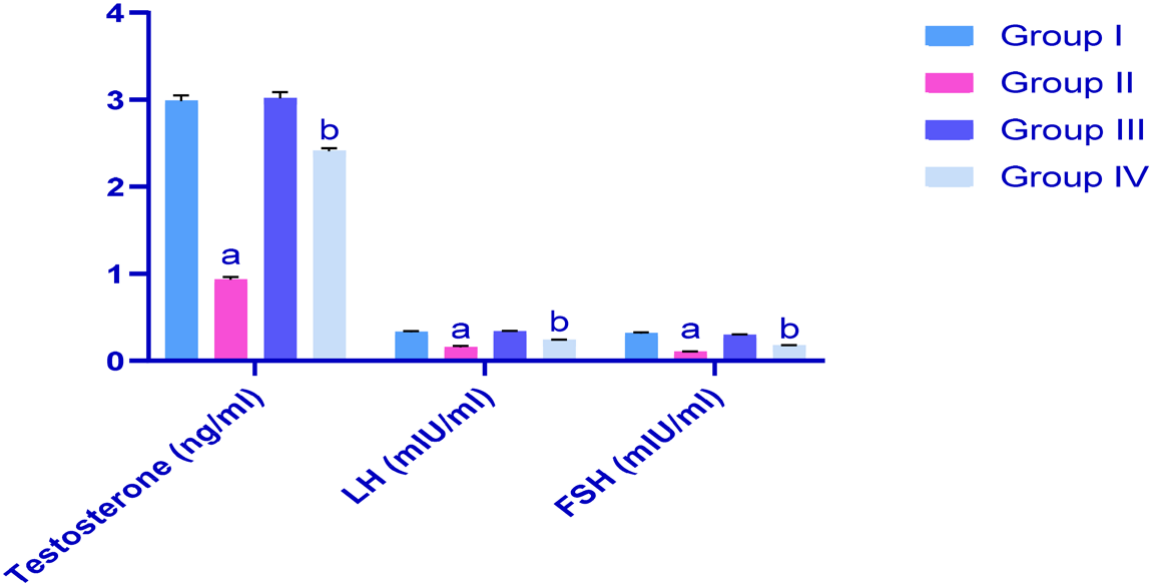
Effect CP treatments on serum levels of testosterone, LH and FSH in rats; G1, control; G2, CP-treated group; G3, IP6-treated group; G4: CP + IP6-treated group; ^a^significant difference compared with groups 1 and 3 (*p* < 0.05); ^b^significant differences compared with G2 (*p* < 0.05). Data are presented as means ± SEM (N = 8), and differences were considered significant when *p* < 0.05.

As shown in Fig. 2, CP had negative effects on rat testicular hormones, as indicated by significant reductions (*p* < 0.05) in testosterone, LH and FSH levels. These hormone levels were improved after treatments with IP6, as compared with rats of G2.

### Effects of treatments on testicle tissue levels of malondialdehyde (MDA), nitric oxide (NO) and total antioxidant activity (TAC) in rat groups

**Figure 3:**
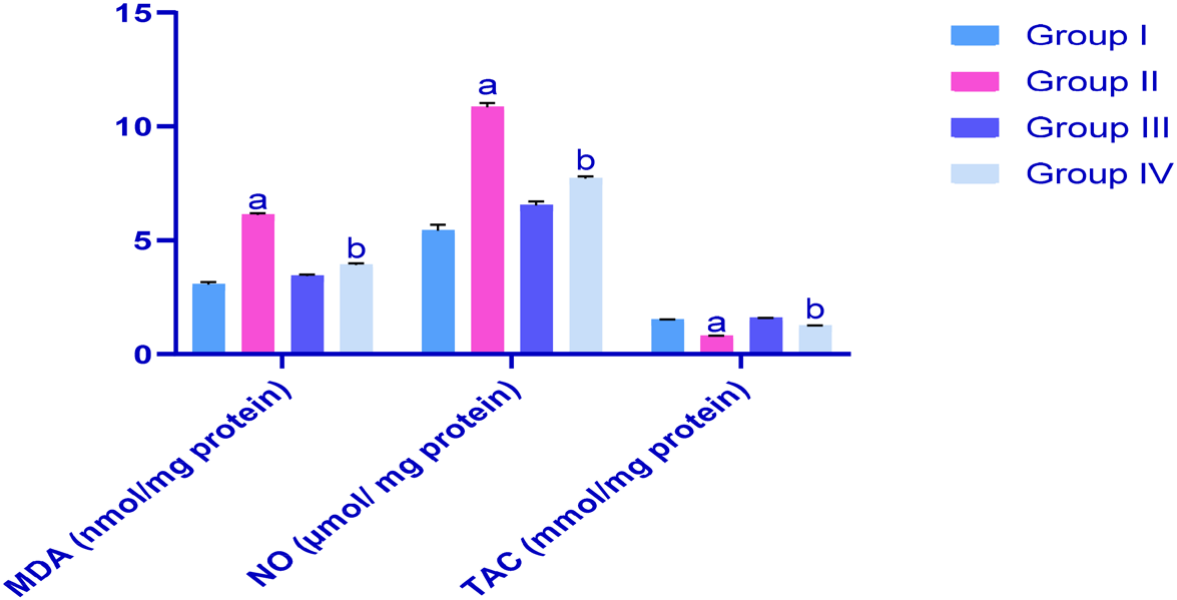
Effects of treatments on testicle levels of MDA, NO and TAC in rats; G1, control; G2, CP-treated group; G3, IP6-treated group; G4 CP + IP6-treated group; ^a^significant differences compared with G1 and G3 (*p* < 0.05); ^b^significant differences with G2 (*p* < 0.05). Data are presented as means ± SEM (N = 8), and differences were considered significant when *p* < 0.05.

As observed in Fig. 3, CP treatments led to significant increases in MDA and NO levels in testicles, with consequent reductions in TAC in G2 rats, as compared with healthy controls. Treatments with IP6 ameliorated this oxidative stress (G4) and restored these parameters to near normal levels.

### Effect of different treatments on testicle levels of alkaline phosphatase (ALP), ACP, GGT and β-glucuronidase in rat treatment groups

**Figure 4:**
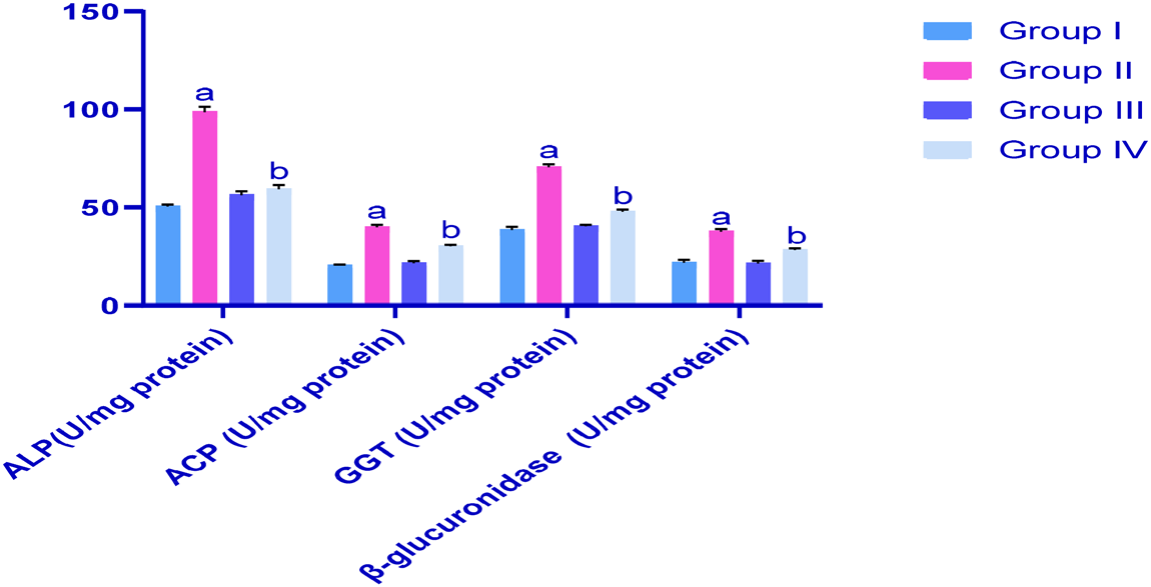
Effects of CP and IP6 treatments on testicle levels of alkaline phosphatase (ALP), acid phosphatase (ACP), gamma-glutamyl transferase (GGT) and β-glucuronidase; ^a^significant differences were observed between G1 and G3 (*p* < 0.05) ^b^and in comparisons with G2 (*p* < 0.05). Data are presented as means ± SEM (N = 8), and differences were considered significant when *p* < 0.05.

Figure 4 shows the effects of different treatments on levels of ALP, ACP, GGT and β-glucuronidase in testicle homogenates from all experimental rats. CP-induced testicle injury is indicated by increased levels of ALP, ACP, GGT and β-glucuronidase in rats of the G2 group compared with healthy control rats of G1. IP6 treatments in G4 moderated increases in levels of these parameters and restored them to near normal levels.

### Effects of CP and IP6 on testicle levels of c-reactive protein (CRP), monocyte chemoattractant protein (MCP-1) and leukotriene-4 (LTB4)

**Figure 5:**
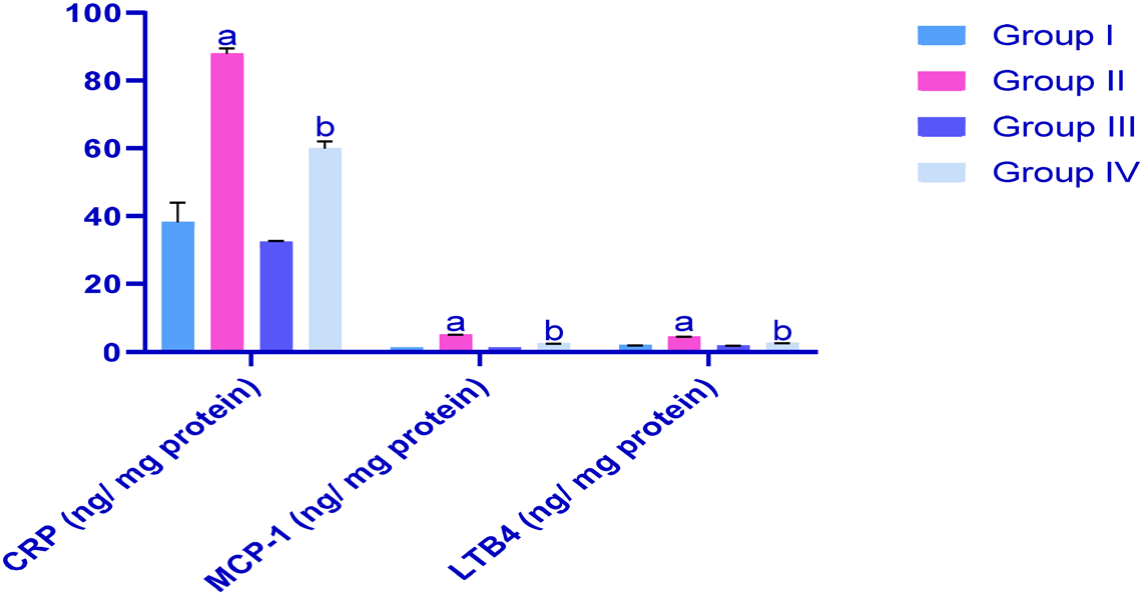
Effects of treatments on CRP, MCP-1 and LTB4 levels in rat testicles; ^a^significant differences were identified between G1 and G3 (*p* < 0.05); ^b^significant differences compared with G2 (*p* < 0.05). Data are presented as means ± SEM (N = 8) and differences were considered significant when *p* < 0.05.

As shown in Fig. 5, CP treatments dramatically increased CRP, MCP-1 and LTB4 levels in testicle homogenates. IP6 attenuated the effects of CP as indicated by comparisons of G4 with G2.

### Effects of CP and IP6 on DNA damage in rat testicular mucosa

**Figure 6:**
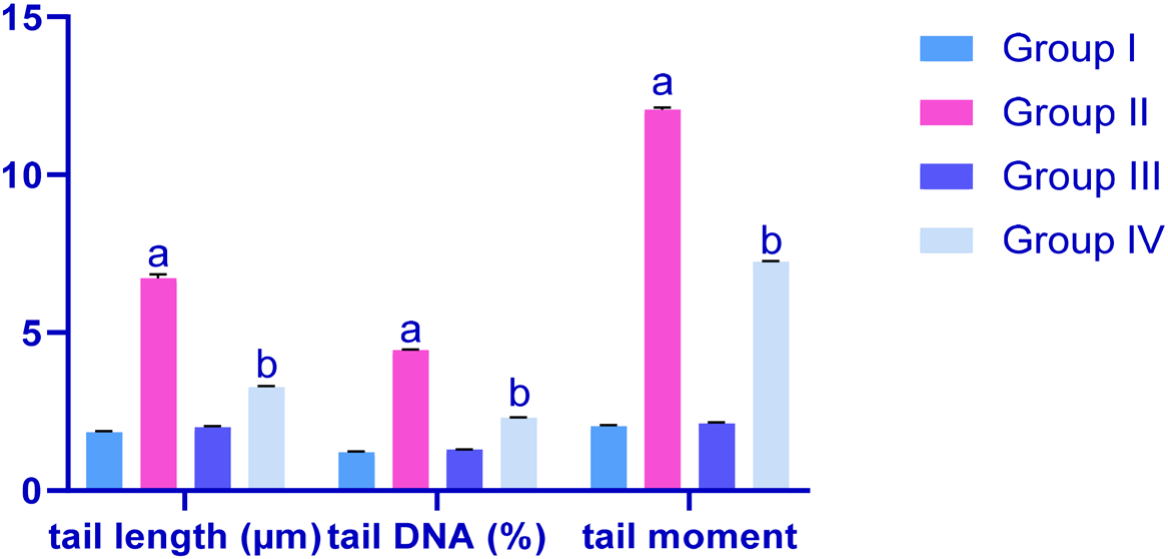
Effects of treatments on DNA damage as assessed by DNA tail length, percentages of tail DNA and DNA tail moments in rats; G1, control; G2, CP-treated group; G3, IP6-treated group; G4 CP + IP6-treated group; ^a^significant differences between G1 and G3 (*p* < 0.05), ^b^significant differences with G2 (*p* < 0.05). Data are presented as means ± SEM (N = 8). Differences were considered significant when *p* < 0.05.

In comet assays, the scoring parameters (Fig. 6) indicated greater DNA damage in testicular mucosa of G3 rats than in G1 rats. Specifically, elongated tail lengths were indicative of increased single-strand breaks and alkali labile sites in testicular DNA. Similar results were obtained for tail lengths and tail moments. In G4, however, concomitant treatments with IP6 inhibited CP-induced DNA damage, with reduced comet tail lengths, % DNA in tails and tail moments, as compared with those in G2.

### Histopathological examinations of testes

**Figure 7:**
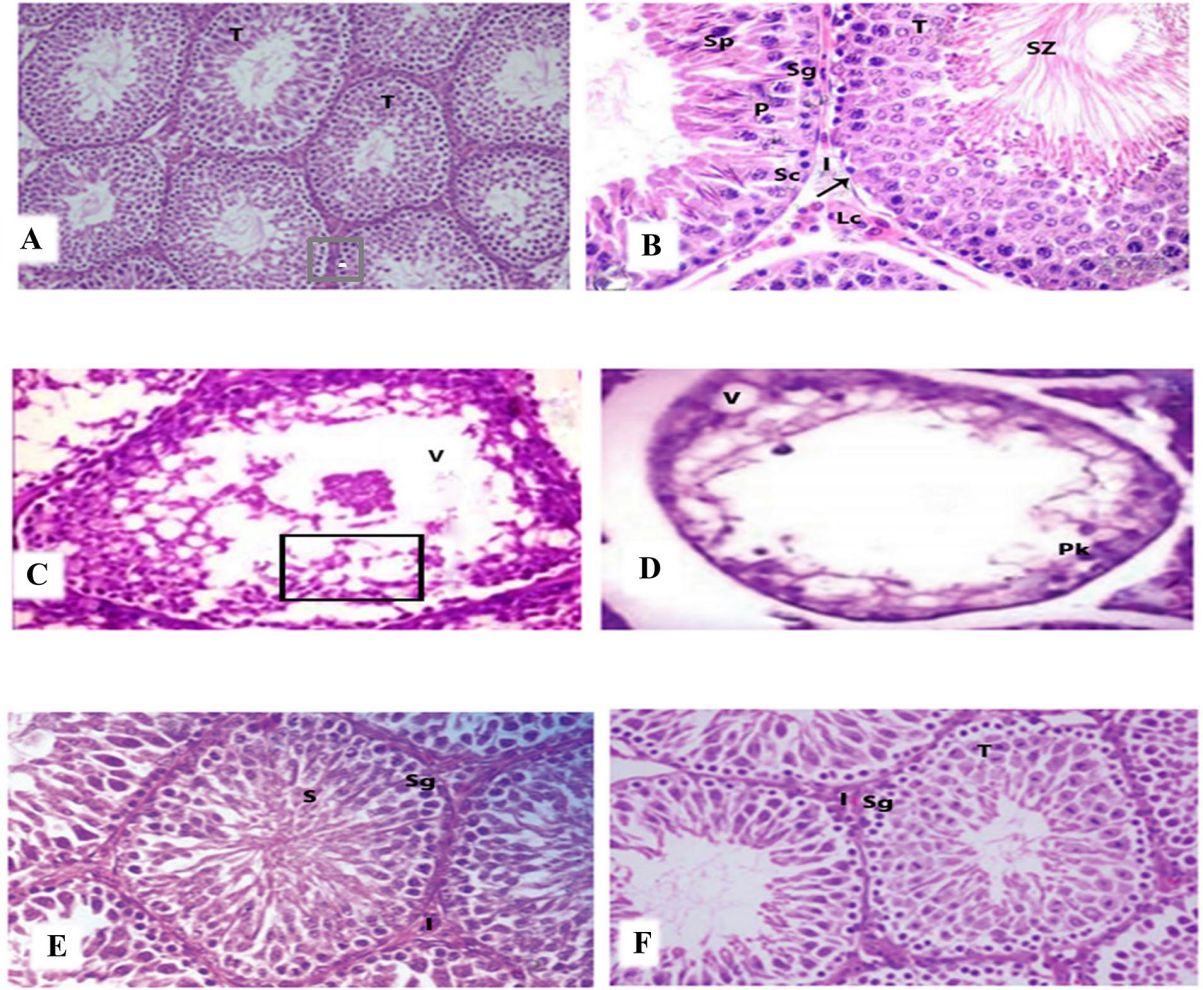
Histological examinations of testis tissue sections; (**A**, **B**) photomicrograph of H&E-stained testis sections in control rats shows normal seminiferous tubules (T) with different types of germ cells; spermatogonia (Sg) on basement membranes (arrow), primary spermatocytes (p) and spermatids (Sp) with normal interstitial tissues (I) and somatic Sertoli cells (Sc). Interstitial tissues between seminiferous tubules contained interstitial cells and Leydig cells (Lc) with acidophilic cytoplasms. The lumen was filled with spermatozoa (SZ; A1, H&E ×200; A2, H&E ×400). (**C**, **D**) A photomicrograph of H&E-stained CP-treated testis showing vacuolation (V), degeneration and necrosis of germ cell linings of seminiferous tubules and oligospermia (B1 H&E 400×). Disorganised germ cells with deeply stained pyknotic nuclei (PK) and cytoplasmic vacuolation (V) and azoospermia (B2 H&E ×400); (**E**) photomicrograph of H&E-stained testis from IP6-treated rats showing normal histological structures in which seminiferous tubules contain normal spermatogenic cells layers and spermatozoa (H&E, ×400); (**F**) a photomicrograph of CP + IP6-treated testis showing remarkable improvements, with closely packed tubular structures and primary spermatocytes, early and late spermatids and large numbers of sperm cells inside the lumen (H&E, ×400).

Histological analyses of testis tissue sections from treated and untreated rats are shown in (Fig. 7A, B). In micrographs of haematoxylin and eosin (H&E) stained sections of control testis, normal seminiferous tubules (T) are visible in different types of germ cells. Spermatogonia (Sg) on basement membranes (arrow) and primary spermatocytes (P) and spermatids (Sp) with normal interstitial tissues between somatic Sertoli cells (Sc) were observed. Additionally, interstitial tissues were observed between seminiferous tubules of interstitial cells, Leydig cells and acidophilic cytoplasms. H&E-stained testis sections from CP-treated rats (Fig. 7C, D), clearly demonstrated vacuolation (V), degeneration and necrosis of germ cell linings of seminiferous tubules and oligospermia. Similarly, disorganised germ cells with deeply stained pyknotic nuclei (PK), cytoplasmic vacuolation (V) and azoospermia were noted in these rats. In contrast, H&E staining of testis sections from rats treated with CP and IP6 showed normal histological structures in which seminiferous tubules contain normal spermatogenic cell layers and spermatozoa were present (Fig. 7E, 40×). Figure 7F, represents CP + IP6-treated testis , a remarkable improvements were seen in closely packed tubular structures with primary spermatocytes and early and late spermatids, and large numbers of sperm cells are visible inside the lumen.

### Sperm DNA damage assessments using comet assays

**Figure 8:**
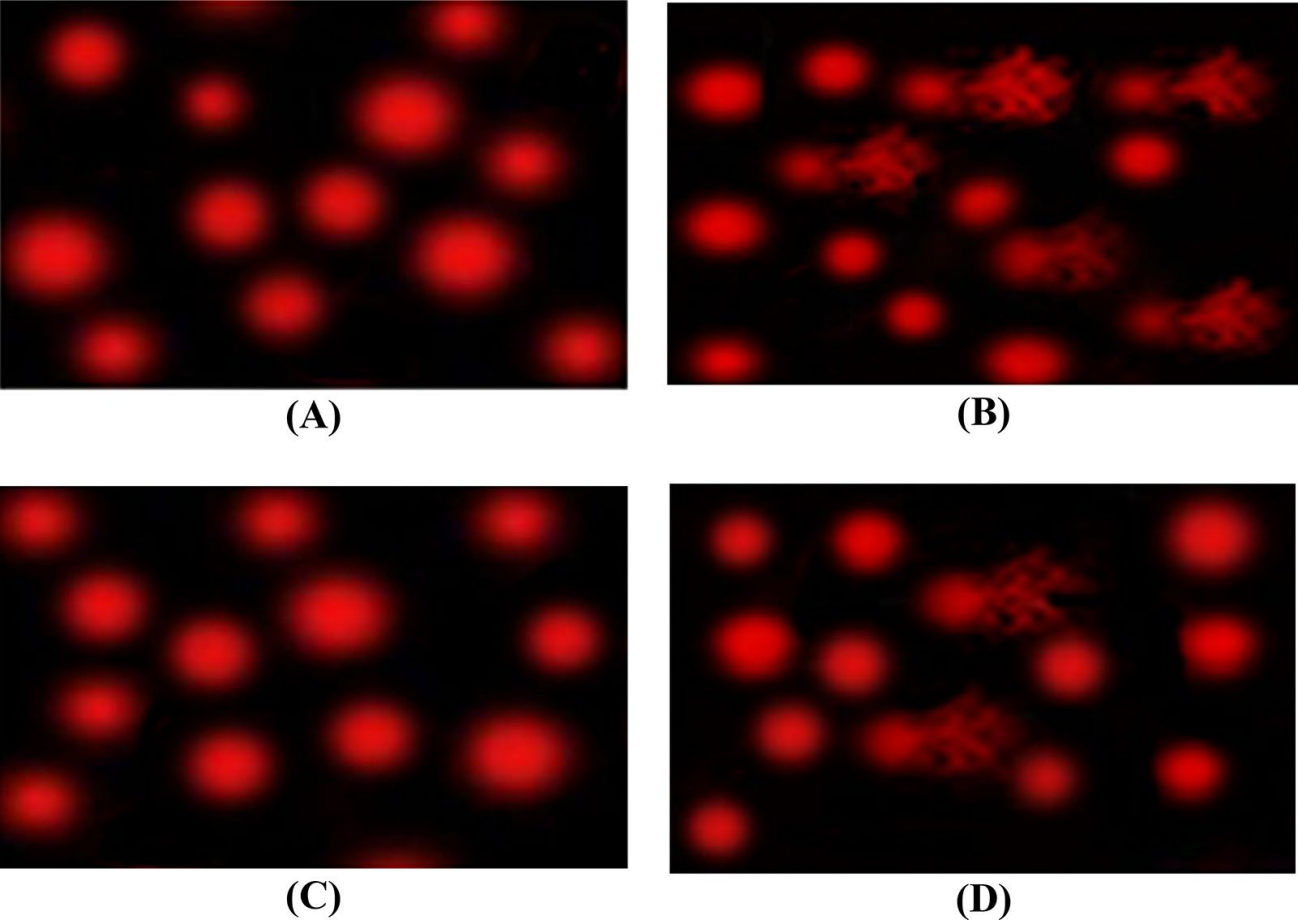
Photomicrograph of comet assay for detection of Sperm DNA damage showing nuclei of testes cells.

Sperm DNA damage assessments using comet assays are shown in (Fig. 8A–D). (A) Photomicrograph of comet assay for normal rats showing normal condensed type nuclei and undamaged cells, (B) Photomicrograph of comet assay for CP-treated rats , showing abnormal tailed nuclei and damaged cells, (C) Photomicrograph of comet assay for IP6-treated rats, showing normal condensed type nuclei and undamaged cells (D) Photomicrograph of comet assay for CP + IP6-treated rats, showing less number of abnormal tailed nuclei and damaged cells.

### Molecular docking analysis

**Figure 9:**
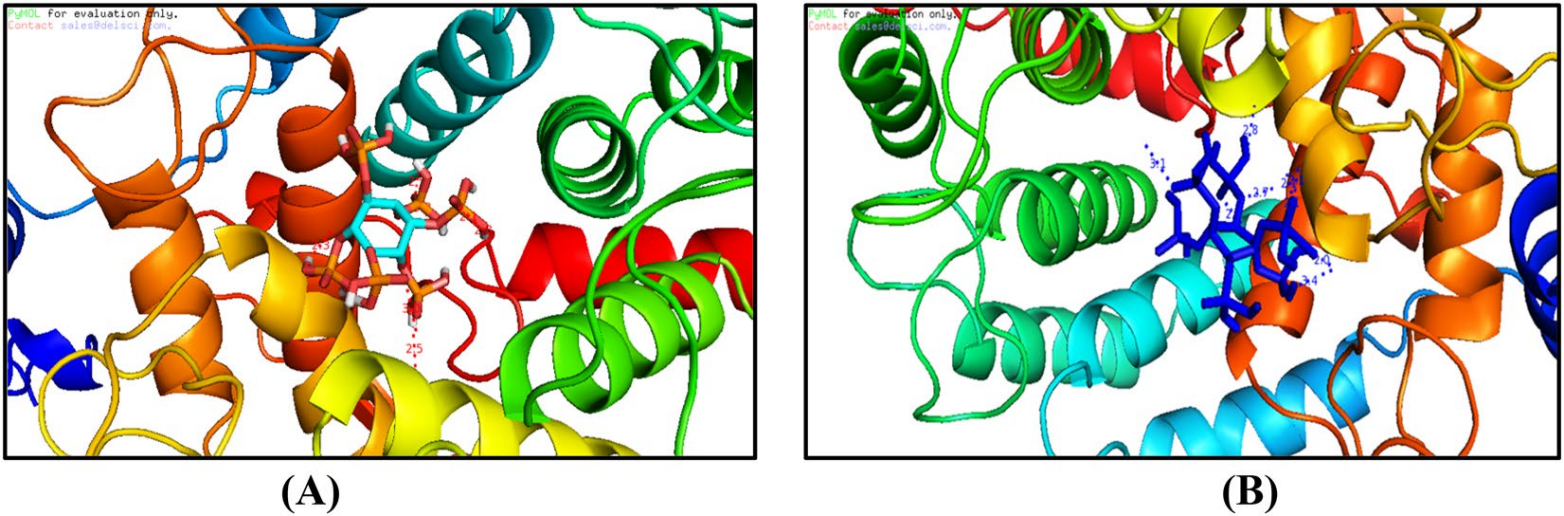
3D plot interaction of Inositol hexaphosphate (IP6) ligand with Testicular-1D8D protein (**A**), and Testicular-1D8E protein (**B**).

**Figure 10:**
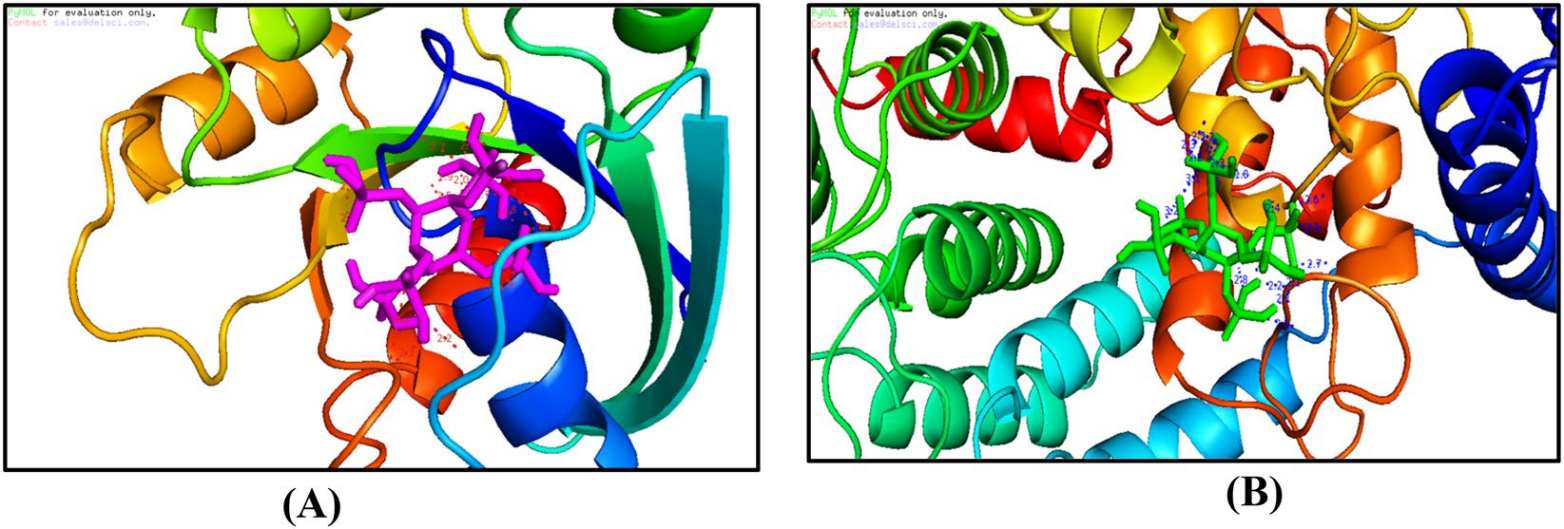
3D plot interaction of Inositol hexaphosphate (IP6) ligand with Testicular-1FLD protein (**A**) and Testicular-1KZO protein (**B**).

We examined antimicrobial properties of the present medicines and experimental agents using auto docking analyses. The guest ligand for four host proteins receptors was IP6-cyclohexane-1,2,3,4,5,6-hexayl hexakis (dihydrogen phosphate) and was examined in molecular docking experiments using Testicular-1D8D protein, Testicular-1D8E protein, Testicular-1FLD protein and Testicular-1KZO protein Figs. 9 and 10. To identify optimal modes of docking, energy and distance data for proteins were submitted to YASARA to reduce protein energy. Similar high-level interactions were observed with all receptors, as shown in 3D plots Figs. 9 and 10. These plots show that multi-central docking ligands have mutable potential for hydrogen bonding interactions^21,22^. Interaction types with molecules in docking pockets were demonstrated using 3D plots Figs. 9 and 10. Hydrogen bonding ligand interactions with amino acids are described below.

1. For the Testicular-1D8D protein, H-bonding interactions with ligands are listed as interactions with amino acids, hydrogen bonds (H-bond) and energy as follows: IP6 ligand with 1D8D protein; 1D8D-B.pdb-h//B/TYR′251/OH (hydrogen bond length = 3 Å),1D8D-B.pdb-h//B/ARG′202/2HH1 (H-bond length = 2.3 Å), 1D8D-B.pdb-h//B/ARG′202/2HH1 (H-bond length = 2.1 Å), 1D8D-B.pdb-h//B/TRP′106/HE1 (hydrogen bond length = 2.5 Å), 1D8D-B.pdb-h//B/TYR′365/OH (H-bond length = 3.3 Å), 1D8D-B.pdb-h//B/TYR′361/OH (H-bond length = 2.3 Å), 1D8D-B.pdb-h//B/GLY′250/O (H-bond length = 2.5 Å) and 1D8D-B.pdb-h//B/TYR′251/OH (H-bond length = 3 Å). The binding energy for these interactions was − 5.51 kcal mol^−1^.
2. For IP6 ligand with the 1D8E-B protein, amino acids, hydrogen bonds and energy were 1-1D8E-B.pdb-h//B/TYR′251/OH (H-bond length = 3.1 Å),1D8E-B.pdb-h//B/TYR′361/OH—(H-bond length = 2.9 Å),1D8E-B.pdb-h//B/GLY′250/HN—(H-bond length = 2.7 Å), 1D8E-B.pdb-h//B/ASP′297/OD2—(H-bond length = 2.4 Å), 1D8E-B.pdb-h//B/ASP′297/OD2—(H-bond length = 2 Å),1D8E-B.pdb-h//B/ARG′291/2HH2—(H-bond length = 2.1 Å), 1D8E-B.pdb-h//B/ARG′291/HE—(H-bond length = 2.1 Å),1D8E-B.pdb-h//B/ARG′291/HE—(H-bond length = 2.1 Å) and 1D8E-B.pdb-h//B/ASP′297/OD2—(H-bond length = 3.4 Å) with a binding energy of − 5.3 kcal mol^−1^.
3. The H-bonding interactions of IP6 ligand with 1FLD-B protein were as follows: 1-1FLD-B.pdb-h//B/LYS′317/HZ1—(H-bond length = 2.7 Å), 1FLD-B.pdb-h//B/LYS′317/HZ1—(H-bond length = 2.3 Å), 1FLD-B.pdb-h//B/ASP′230/OD1—(H-bond length = 2.1 Å), 1FLD-B.pdb-h//B/ASP′230/OD1—(H-bond length = 3.4 Å), 1FLD-B.pdb-h//B/LYS′231/HN—(H-bond length = 2.2 Å), 1FLD-B.pdb-h//B/SER′217/HG—(H-bond length = 3.2 Å),1FLD-B.pdb-h//B/ASP′233/HN—(H-bond length = 2.2 Å), 1FLD-B.pdb-h//B/ASP′233/HN—(H-bond length = 2.7 Å), 1FLD-B.pdb-h//B/SER′217/OG—(H-bond length = 3.2 Å),1FLD-B.pdb-h//B/GLY′213/HN—(H-bond length = 2.2 Å), 1FLD-B.pdb-h//B/SER′217/HN—(H-bond length = 1.8 Å), 1FLD-B.pdb-h//B/LYS′216/HN—(H-bond length = 2 Å),1FLD-B.pdb-h//B/LYS′216/HN—(H-bond length = 2.7 Å), 1FLD-B.pdb-h//B/VAL′214/HN—(H-bond length = 2.7 Å) and 1FLD-B.pdb-h//B/GLY′215/HN—(H-bond length = 2.5 Å), with a binding energy of − 6.5 kcal mol^−1^.
4. The H-bonding interactions of IP6 ligand with 1KZO-B protein were as follows: 1-1KZO-B.pdb-h//B/LYS′294/HZ1—(H-bond length = 2.4 Å), 1KZO-B.pdb-h//B/LYS′356/HZ3—(H-bond length = 2.5 Å), 1KZO-B.pdb-h//B/ASP′352/OD2—(H-bond length = 2.1 Å), 1KZO-B.pdb-h//B/ASP′352/OD2—(H-bond length = 2.1 Å), 1KZO-B.pdb-h//B/HIS′362/HE2—(H-bond length = 2.8 Å), 1KZO-B.pdb-h//B/ASP′297/OD2—(H-bond length = 2.7 Å), 1KZO-B.pdb-h//B/TYR′361/OH—(H-bond length = 3.2 Å), 1KZO-B.pdb-h//B/TYR′300/OH—(H-bond length = 3.1 Å), 1KZO-B.pdb-h//B/TYR′300/HH—(H-bond length = 2.5 Å), 1KZO-B.pdb-h//B/TYR′300/OH—(H-bond length = 2.5 Å), 1KZO-B.pdb-h//B/HIS′248/HE2—(H-bond length = 2.2 Å), 1KZO-B.pdb-h//B/HIS′248/HE2—(H-bond length = 2.3 Å), 1KZO-B.pdb-h//B/ARG′291/HE—(H-bond length = 2.5 Å), 1KZO-B.pdb-h//B/ARG′291/1HH2—(H-bond length = 2.2 Å), 1KZO-B.pdb-h//B/LYS′294/NZ—Length (H-bond length = 2.5 Å) and 1KZO-B.pdb-h//B/LYS′294/HZ1—(H-bond length = 2.4 Å), with a binding energy of − 5.1 kcal mol^−1^.

The results above indicate binding of IP6 to amino acids for four selected proteins. IP6 binding of amino acids was suggested by numbers of hydrogen bonds, small root-mean-square deviation distances and high binding energy, indicating that IP6 releases toxic materials from proteins and thereby reduces toxicity. IP6 was also located in cavities of native ligands that were linked to proteins.

## Discussion

Some chemotherapy drugs, especially alkylating agents, are genotoxic and are associated with reproductive toxicity pending on their antineoplastic activities, overall dosages, durations of treatment and individual sensitivities^23^ Because of their high mitotic activity, spermatogenic cells are targeted by these alkylating agents. Therefore, strategies for reducing the side effects of cancer medicines and maintaining their chemotherapeutic efficacy are eagerly awaited.

Testicular toxicity is severely limiting the efficacy of CP as an anti-cancer and immunosuppressive therapy^24^. Accordingly, reproductive organ weights and fertility rates are decreased in male rats receiving CP. Under these conditions, CP is considered a chief cause of dysfunctional spermatogenesis^25^. CP therapy has also been shown to significantly decrease sperm motility and viability and to cause changes in testicle histology. Evaluations of sperm and testosterone levels are core to assessments of testicle damage. CP induces injury during the early phases of spermatogenesis, particularly in testicular cells^26^. The data presented here indicate that addition of IP6 to drinking water is protective against the deleterious effects of CP on testicle function.

The integrity of steroidogenic and spermatogenic pathways is related to sperm counts and consistency. Oxidative stress decreases circulating testosterone and LH levels because of inhibition of 17-ß hydroxysteroid dehydrate and 3-ß hydroxysteroid dehydrogenase enzyme activities^27^. Potentially, failure of spermiogenesis in CP-treated males follows disruptions of testosterone-based relationships between Sertoli and germ cells, which contribute to their breakup and breakout. Moreover, decreased sperm counts were reportedly associated with loss of sperm cells at various stages of development, and these effects were related to free radical attack^28^. Testicle tissues are very sensitive to ROS, which cause oxidative damage to polyunsaturated fats in mammalian sperm membranes, likely leading to increased membrane permeability and damage of germ cells, spermatozoa and mature sperm^29^.

The effects of IP6 on CP therapy-induced pathological alterations were previously elucidated in terms of critical cellular functions, such as cell proliferation and anti-cancer properties. In a review of IP6, improved reproductive functions were cited in many studies^30^. We show that IP6 significantly decreases the adverse effects of CP on hormones like testosterone, LH and FSH, and these effects are similar to those reported previously^24^. In particular, testosterone is susceptible to sustained CP regimens, along with bodyweights and sex organs. After oral administration, CP is metabolised into phosphoramide and acrolein by hepatic cytochrome P450. Acrolein suppresses the synthesis of sex hormones and induces sperm cell apoptosis, thus affecting fertility. Decreased plasma testosterone levels may also follow impaired spermatogenesis in CP-treated rats, as suggested in the present study. In addition to hormone changes, CP may inhibit spermatogenesis because of increased free radical production in testicle tissues^31^.

Lowered testosterone production and defective androgen receptors following CP treatments may impact testicle functions by affecting sperm and increasing the prevalence of morphological sperm defects^32^. With LH, which is secreted by the pituitary gland^33^, FSH targets ovarian granulosa cells in females and seminiferous tubules in males. It promotes follicular development and ripening, enhances quality and secretion of estrogens and increases the growth of sperm and seminiferous tubules^12^. In combination with FSH, androgens activate protein production and various other factors that are important for germ cell differentiation^34^. Among potentially bioactive substances in soy meal, however, the effects of IP6 on metabolism of sex steroids and hormonal activity are challenging to demonstrate^35^. Yet IP6 improved the effects of aflatoxin B1 injections on pathological and hormonal changes^36^.

Oxidative stress is a primary cause of male infertility and peroxidative damage under various stress conditions is widely known as the main cause of testicle injury. Among related mechanisms, increased oxidative stress in testicles was previously associated with changes in microvascular blood flows that increase germ cell apoptosis^37^. Increased MDA and NO levels and related decreases in TAC levels in testicular homogenates are often used as proxies for oxidative stress in cells. Herein, CP increased oxidative stress, and ROS mediated testicular injury^38^. Similarly, earlier studies associated CP with increased ROS production, oxidation of biological membranes and MDA levels and reduced natural liver tissue antioxidant defences^39^. These observations of acrolein effects are mainly indicative of interference with protective antioxidant systems. In addition to its harmful effects on male reproductive function^40^, acrolein induces apoptosis in testicle tissues, thus undermining male fertility and lowering testosterone levels, particularly in younger patients^41^. As indicated by the present data, the resulting increases in NO intensify inflammatory responses after testicle damage^42^.

Our results show that administration of IP6 in drinking water increases oxidative/nitrosative status and restores enzyme activities almost to normal levels. A previous study suggests that IP6 is a natural antioxidant that protects against disease and prevents the development of tumors^36^. Moreover, IP6 is known to suppress Fenton reactions and the subsequent release of hydroxyl radicals by chelating cations of iron. IP6 also reportedly inhibited the development of ROS in biological tissues, thereby protecting against free radical damage in cells and tissue following inflammation, hypoxia or exposure to radiation. In addition, scavenging of superoxide radicals prevents the formation of complexes of ADP and iron that cause lipid peroxidation in vivo and in vitro. Accordingly, IP6 was shown to inhibit lipid peroxidation in previous animal studies^43^.

Herein, we show significant increases in ALP, ACP, GGT and ß-glucuronidase activities due to CP-induced testicular injury. ALP plays important roles in spermatogenesis and is vital to the survival and motility of sperm, and its activity was previously correlated with testicle degeneration^44^. Similarly, ACP activities are diagnostic of prostate cancer metastases and are often used to assess treatment efficacy. Prostate tissues also have high acid phosphatase activities. Hence, elevated ACP activities are used as a biomarker of damage to the prostate gland. ACP is also involved in intracellular digestion of endogenous and phagocytosed exogenous phosphate residue-containing compounds. These participate in the penetration of sperm through eggs in the acrosome^45^. In mammals, GGT degrades oxidised and reduced glutathione at cell surfaces by breaking gamma glutamate bonds. This enzyme is predominantly expressed in the caput epididymis^46^. In proximal epididymal regions, GGT activities are higher than in distal regions^47^. During sperm ripening, GSH also participates in peroxidation following metabolism by GGT and acts as a major antioxidant^48^.

We also determined activities of ß-glucuronidase, because it is a major phase I metabolising enzyme of the lysosomal glycosidase family and is distributed widely in various mammalian tissues^49^. In tumour cells of colon carcinomas, liver cancers and prostate cancers, ß-glucuronidase activities are demonstrably higher than in normal cells. Hence, in clinical applications, ß-glucuronidase has been recognised as a potential tumour biomarker^50^.

Elevated activities of the enzymes described above were disrupted by IP6 in our rat experiments and were almost restored to normal levels. Accumulated data from animal disease models suggests that IP6 supplements can provide substantial protection against colon cancers. Furthermore, IP6 regulates the numbers and growth rates of cells by preventing cell division and thereby prevents overwhelming of the immune system. In laboratory and animal studies, inositol slowed the spread of cancers. Because increases in lipid peroxides and ROS have been associated with cancer development, the antioxidant activity of inositol may contribute to the anti-cancer effects of its derivatives Inositol-6-phosphate and Myo Inositol^30^.

In previous studies, IP6 had a positive impact on human prostate cancers^51^ and on prostate cancer cells in vitro^52^. Moreover, dose-dependent growth inhibition and suppression of DNA synthesis were observed in male prostate cancer cells that were treated with IP6 (phytic acid). Significant increases in activities of prostatic acid phosphatase were also observed in the presence of IP6, and this enzyme is a precursor for prostatic cell differentiation^53^. In agreement with our study, a chemo-preventative effect of 2% IP6 in drinking water was shown in animal models of chronic disease^54^, further demonstrating that IP6 inhibits cell proliferation and induces apoptosis. Another study^55^ showed decreased incidence and multiplicity of colon cancers in rats after treatments with IP6, and these effects on cancer cells were related to mechanisms involving gene modifications, immune enhancements, antioxidant properties and metal chelating ability^56^. CP treatments dramatically increased CRP, MCP-1 and LTB4 levels in testicle homogenates. Moreover, CP metabolites are highly toxic towards sinusoidal endothelial cells and, after production in hepatocytes, are transported into hepatic sinusoids. Subsequent death of endothelial cells causes endothelial vascular dysfunction, which leads to inflammatory reactions by enhancing the interactions between leukocytes and endothelial cells^57^.

CRP is a well-studied, non-specific marker of inflammation and is mainly synthesised by hepatocytes^58^. Furthermore, high levels of CRP are most commonly seen in patients with testicular cancers^59^. As a prominent monocyte-selective cytokine attractant protein, MCP-1 is produced by several interstitial testicular cell types in vitro. Because this protein attracts inflammatory cytokines, MCP-1 may recruit monocytes in vivo^60^. LTB-4 is an arachidonic acid metabolism-related lipoxygenase found predominantly in polymorphonuclear, mononuclear and epithelial cells. It is also known as a potent proinflammatory lipid mediator that is synthesised by immune cells and stimulates secretion of multiple cytokines. Neutrophil activation is often used as an indicator of LTB-4, which is associated with growth of cancer cells^61^. In contrast, IP6 reduced proliferation and induced apoptosis and differentiation of many types of malignant cells. Moreover, proinflammatory cytokine levels were reportedly reduced by IP6^62^.

In support of our observations, CP treatments previously caused DNA damage in sperm and decreased sperm counts and motilities in rats^63^. Additionally, when administered at 5 mg/kg/day for 28 days, CP induced atrophy of seminiferous tubules and extreme sperm cell shortages, thus greatly impacting fertility^64^. Furthermore, when formed in large quantities, ROS causes DNA fragmentation and sperm degradation related to mitochondrial peroxidative damage and losses of sperm membrane integrity. Sperm are particularly vulnerable to peroxidative damage because they have high concentrations of polyunsaturated fatty acids and poor antioxidant potential^63^. CP is a DNA alkylating agent that prevents DNA replication during cell division, leading to cell cycle arrest at the S phase and induction of apoptosis in telencephalon embryonic neural progenitor cells at 6–12 h after administration^65^.

The anticancer mechanisms of IP6 may relate to antioxidant properties, immune-enhancing functions and regulatory effects on signal transduction. Through these related pathways, IP6 controls cell cycle progression, apoptosis and cell proliferation^66^. Moreover, IP6 supplementation inhibits molecular targets of inflammation, angiogenesis and metastasis^67^.

Finally, decreased synthesis of testosterone following long-term exposure to CP has been correlated with histological changes. Following administration of CP to rats, atrophy of seminiferous tubules with interstitial vacuolation may decrease sperm viability and motility, as reported in multiple studies^24,26^. CP was also shown to increase the absorption of testicular haemoglobin, which is a hallmark of oedema and haemorrhage, as indicated by histopathological analyses^8^. These effects reflected associations of CP with proteins containing sulfhydryl groups. Furthermore, excessive ROS generation likely damages blood vessels directly^68^.

## Conclusion

In this study, we generated data showing that CP disrupts redox balance in testicular tissues and thereby impairs testes functions by adversely disturbing sperm properties, hormonal levels and testes histology. CP also impairs oxidative states, increases inflammation and affects immunity. Importantly, we show that IP6 in drinking water ameliorates these harmful effects of CP on reproductive function. Hence, for cancer patients receiving CP treatments, IP6 can be considered an effective medical treatment for avoiding male infertility.

## Material and methods

### Chemicals

CP was obtained from Endoxans (Baxter Oncology, Germany), and IP6 was purchased from Sigma Chemical Company (St. Louis, MO).

### Experimental animals

Methods were carried out in accordance with approved guidelines. All experiments were performed per the National Institutes of Health Guiding Principles within the Care and Use of Animals. Forty adult male Albino rats weighing 150–170 g were housed at the animal facility of the King Fahd Medical Research Center and were used in experiments after a 1 week acclimation period at 24 °C ± 1 °C in 45% ± 5% humidity with a 12 h light/12 h dark cycle. During the experimental period, a commercially balanced diet and tap water were provided ad libitum. The experiment was approved by the Ethical Committee of King Fahd Medical Research Center. Jeddah, KSA. Approval number (172-19).

### Experimental design

Rats were divided into four groups (10 each) as follows: G1, healthy rats (negative control); G2, rats with testicular toxicity (positive control) due to single i.p. injections of CP (200 mg/kg of body weight); G3, rats supplemented with 2% IP6 (w/v) in drinking water for 2 weeks and G4, rats receiving i.p. injections of CP and supplements with 2% IP6 (w/v) in drinking water for 2 weeks. In All groups supplemented with tested agent daily for 2 weeks, sedative or toxic effects were tested in all rats. The rats were then sacrificed by cervical dislocation under diethyl ether anaesthesia. Blood samples were collected from the retro-orbital venous plexus in all animals and were placed in heparinised tubes for biochemical assessments. Testes were collected through incisions in the lower abdominal area and were then thoroughly dissected. Appropriate tissue samples for histopathological analysis were processed for light microscope and electron microscope analyses. The remaining samples were instantly frozen in liquid nitrogen and were partly divided into pieces. These were stored at − 80 °C until preparation of tissue homogenates.

### Evaluations of sperm counts, motility, viability and abnormalities

Cauda epididymis tissues from all rats were crushed and carefully mixed with 10 ml aliquots of 0.9% NaCl. Mixtures (20 µl) were then transported to a haemocytometer in Mallassez and numbers of sperm/ml were counted under a light microscope (OLYMPUS, 400)^3^. At 1–2 min after sacrifice, sperm motility was determined. Percentage of sperm motility was calculated using the following formula:

$$ {\text{Motile}}\,{\text{sperm}}\,\left( {\text{\% }} \right) = {\text{N}}/{\text{T}} \times 100 $$

where N is the number of motile sperm, and T is the total number of counted sperm.

For sperm viability analyses, 10 µl diluted epididymal tissue samples were combined with 10 ml aliquots of eosin (5 mg/ml) and 100 ml of nitrogen (1 mg/ml) and were then placed on slides and gently oven dried. The unstained sperm were identified as viable. A total of 200 sperms/slide were counted and percentage sperm viability was calculated as previously described^3^. Samples in Hancock solution were fixed, and smears were then prepared to identify sperm abnormalities. After staining slides with Giemsa's dye, smears were analysed at 400× magnification. A total of 200 spermatozoa were analysed from each rat and were scored individually as normal or abnormal according to strict sperm morphology benchmarks. Morphological aberrations were categorised as defects in head and tail parts. As part of the examination of the sperm tails, sperm anomalies in the mid-piece were included. Percentages of normal and abnormal sperm were determined as described previously^17^.

### Preparation of testicle homogenates

Testes samples were excised, dissolved, desiccated, weighed and homogenised in double-distilled ice-cold physiological saline. Appropriate aliquots from various homogenates were added to Tis–HCl buffer (0.01 mol/L, pH 7.4) and were centrifuged at 10.000×*g* at 4 °C for 30 min to obtain 10% homogenate solutions.

### Biochemical determinations

#### Serum

Levels of testosterone, LH and FSH in rat serum were assessed using Randox kits and enzyme-linked immunosorbent assays (ELISA) according to the manufacturer’s instructions^18^.

### Testicular homogenates

MDA, NO and TAC were spectrophotometrically evaluated using Bio-vision Kits (CA, USA). ALP, ACP and CRP levels were determined using ELISA kits (Kamiya Biomedical Co., CA, USA). GGT was detected using an ELISA kit from Abcam (OR, USA), and B-glucuronidase was estimated using an ELISA kit from Syntron (Bioresearch, Inc., CA, USA). Similarly, MCP-1 and LTB4 levels were determined using ELISA kits from Mybioscience (CA, USA) following the manufacturer’s instructions.

### Histopathology examinations of testes

In 10% formalin, testicle tissues were fixed and dehydrated and then coated in paraffin. Tissues were then cut into 5 μm-thick deparaffinised sections, which were then stained with H&E.

### Sperm DNA damage assessments using comet assays

To assess DNA damage using one-cell electrophoresis techniques (comet assay), 0.5 g crushed samples were suspended in 1 ml aliquots of ice-cold PBS. Cells were then combined with 600 µl aliquots of low melting agarose (0.8% in PBS). Subsequently, 100 μl mixtures were dispersed over fully frozen microscope slides precoated with 0.5% standard agarose at its melting point. Coated slides had been soaked in solutions containing 0.045 mol/L Tris/Borate/EDTA (pH 8.4) and 2.5% sodium dodecyl sulphate for 15 min. Slides were then placed in electrophoresis chambers and were electrophoresed at 2 V/cm and 100 mA for 2 min. After washing in buffer containing 0.4 M tris hydrochloride (pH 7.5) for neutralisation, slides were treated with 20 μg/ml ethidium bromide at 4 °C. Ethidium bromide-stained DNA damage was then viewed with excitation at 420–490 nm using a 40× objective lens and a fluorescence microscope connected to camera (Olympus). DNA damage was assessed using a computer-based imaging system according to olive tail moments and percentages of DNA in the tails (percent tail DNA) of 50 cells per sample.

### Molecular docking

Docking computations were performed using the Auto Dock 4.2 module with Gasteiger’s partial charges attached to atoms of designed drug ligands programe. Calculations were achieved with ligand–protein patterns. Non-polar hydrogen atoms were joined together to explain rotatable bonds. Kollman fused atomic charges with recovery parameters were calculated after adding central hydrogen atoms using AutoDock^19^. Vanderwaal and electrostatic bonds were determined using parameter set- and dielectric distance-dependent functions, respectively. Simulative docking was performed using the Solis and Wets local procedure and the Lamarck genetic algorithm. Ligand molecules were identified in initial positions, orientations and torsions. Upon docking, all rotatable torsions were removed. Each docking experiment was blocked following an ultimate energy estimate of 250,000 in 10 separate runs^20^.

### Statistical analysis

Statistical analysis was performed using the Statistical Package for the Social Sciences (SPSS programme for Windows, version 20; SPSS Inc., Chicago, IL, USA). The results were expressed as the mean ± standard error. The differences between mean values were determined by using the one-way analysis of variance (LSD) test. A *p* value < 0.01 was considered significant.

## Data Availability

The data used during the current study are available from the corresponding author.
